# Virtual Teams in Times of Pandemic: Factors That Influence Performance

**DOI:** 10.3389/fpsyg.2021.624637

**Published:** 2021-02-17

**Authors:** Victor Garro-Abarca, Pedro Palos-Sanchez, Mariano Aguayo-Camacho

**Affiliations:** ^1^School of Computing, Tecnológico de Costa Rica, Cartago, Costa Rica; ^2^Department of Financial Economics and Operations Management, University of Seville, Seville, Spain

**Keywords:** global software development, COVID-19, virtual teams, determinants of performance, PLS-SEM

## Abstract

In the digital age, the global software development sector has been a forerunner in implementing new ways and configurations for remote teamwork using information and communication technologies on a widespread basis. Crises and technological advances have influenced each other to bring about changes in the ways of working. In the 70’s of the last century, in the middle of the so-called oil crisis, the concept of teleworking was defined using remote computer equipment to access office equipment and thus avoid moving around using traditional vehicles. Then from the 90s, with the advent of communications and the widespread use of the Internet, the first virtual work teams were implemented in software development companies that already had some of the important characteristics needed to work in this way, such as, cultural diversity, characterized tasks, geographical distribution of members, communication, interdependence of tasks, leadership, cohesion, empowerment, confidence, virtuality. This manuscript groups the main factors into different models proposed by the literature and also analyzes the results of a study conducted in the midst of the Covid-19 crisis on 317 software development teams that had to work in virtual teams (VT). The results of the quantitative methodology with structural equation modeling based on variance using the partial least squares route method are analyzed. The results of the research focus on some determinants that can directly affect the performance of the virtual team. A first determinant is communication in relation to the tasks. The second is trust in relation to leadership, empowerment and cohesion. The results of virtual teams provide information that can serve as a basis for future research lines for the implementation of virtual work strategies in post-pandemic work.

## Introduction

The digital era has meant a change in the processes and routines of the business dynamics to which many organizations have had to adapt in order to compete and survive in globalized markets. The virtualization of organizational life and the digital transformation of labor relations goes hand in hand with the accelerated advance of technologies such as cloud computing, which have made it unnecessary to have tangible servers, software and hardware infrastructures in the company offices and many processes are being carried out by accessing personal equipment or terminals (computers, laptops, and mobile devices) connected to an increasingly fast Internet network. All this is possible thanks to the technology of virtualization (Sánchez, 2017). Recent studies have analyzed the attitude of human resources to cloud technology and its importance in software as a service application - SaaS- (Palos and Correia, 2017) and how the attitude of the worker has changed, thanks to online work training (Palos-Sanchez, 2017). Thus, the digital virtualization of traditionally physical technological resources is also happening at the level of human resources, because increasingly the presence of workers in the same place is not necessary. This implies an immense challenge for the new electronic leadership of teams of collaborators who are increasingly dispersed geographically.

In the beginning, virtual teams were formed to facilitate joint creation and innovation among global or regional experts who did not have enough time to travel to fulfill the specialized tasks of the projects that required them. Today, virtual teamwork has evolved to a point where online collaboration is a way of working for national companies and more naturally for multinational or regional companies. The idea of virtual collaboration between workers, or virtual teamwork VT, consists of a team working together from different physical locations using collaborative ICTs. In the last 20 years this modality has been in constant growth due to the evolution and maturity of the digital era in terms of speed of telecommunications, the power of the computer equipment, the naturalness of adaptation to the use of ICTs in the work of digital natives (born since 1990) and digital migrants (born before 1990). However, at the beginning of the 21st century it was difficult to have faith in VTs due to the low level of maturity of virtual teams which made companies skeptical about the efficiency of this way of working. By the early 2000s, studies showed that the number of VTs that achieved their goals was not very encouraging and there was a significant failure rate. A few years later, things had not changed that much either. In 2004, there was talk of significant challenges in the implementation of virtual teams (Piccoli et al., 2004). Another study (Brett et al., 2006) revealed that most people thought that virtual communication was not as productive as face-to-face interaction, while half of the respondents said they were confused and overwhelmed by collaboration technology. Even so, this happened a few years ago and as technology advanced, companies matured with the use of ICT tools, so these early conclusions from the beginning of the century were not believed to be accurate anymore. A more recent study in 2009, involving 80 global software teams, indicated that well-managed virtual teams using virtual collaboration can outperform face-to-face (FtF) teams.

Additionally, a number of studies (Jarrahi and Sawyer, 2013), indicate that virtual or remotely distributed team collaboration can also improve employee productivity. Therefore, an important question is: what can make a virtual team have better performance results than a face-to-face team? The answer has been provided by several studies that have summarized input factor models and their relationships with other factors grouped into socio-emotional and task-oriented processes and finally their relationships with output factors (Powell et al., 2004; Gilson et al., 2015).

In addition to the aforementioned triggers of virtualization of organizational life and the digital transformation of processes (Zúñiga Ramirez et al., 2016) and the interrelations of stakeholders as co-creators of value (Martinez-Cañas et al., 2016; Ribes-Giner et al., 2017), it is also worth mentioning that the origin of remote work in a virtual team is originally teleworking.

Considering the above reasons and in view of finding ourselves in the midst of a rapidly evolving digital era coupled with a pandemic that has forced workers in many areas to perform remote work (Velicia-Martin et al., 2021) and aligned with an effective strategy to contain and mitigate rate of spread of infection (Brooks et al., 2020), this study has been undertaken in the midst of the COVID19 impact on virtual teams in the software development industry. The co-creation in virtual teamwork is a very important feature.

The main objective of this research, at a time with a pandemic and the current digital era (Chen et al., 2020), is to analyze the relationship of important factors found in the literature by analyzing the performance of 317 software engineers in virtual teams. Software engineers, due to their training and experience, belong to virtual teams that include co-creation for the construction of software using agile methodologies and have recently been involved in working in virtual teams. This research is original because of the importance given to endogenous variables such as communication and trust. For this reason, the results of the survey carried out have served to understand what role different factors play in the performance of a group used to doing remote or virtual teamwork as part of their normal work. The study uses a structural equation approach with partial least squares (PLS) to evaluate the proposed performance model. The research is organized as follows. First, the Introduction explains the article based on the history of co-creation in current software development and its relationship to the study of vital equipment. Then there is a literature review, which analyzes relevant research on factors in VTs. Thirdly, methodology and justification of the hypotheses are presented. The results are then analyzed. In the Conclusions section, discussions and conclusions are made in which the practical implications of the research are given.

## Literature Review

A virtual team is defined as a group of people or stakeholders working together from different locations and possibly different time zones, who are collaborating on a common project and use information and communication technologies (ICTs) intensively to co-create. It can be seen that one of the main characteristics is virtuality, which implies physical and temporal distance between members and a shared purpose (Ebrahim et al., 2009).

Another essential characteristic of virtual teams, which differentiates it from traditional “face-to-face” (FtF) teams is the collaborative use of technology for work. This has been the result of the evolution of ICTs in this digital age, along with the trend toward globalization. In VTs there is naturally a geographical dispersion that entails certain cultural differences and social bonds are more difficult to achieve. All this generates a series of difficulties for communication between members and emotional relationships (Duarte and Snyder, 2006; Lin et al., 2008; Shuffler et al., 2010).

Virtual teams are affected by a series of factors and phases, which have been investigated in the literature (Abarca et al., 2020) and which give rise to different models for studying and relating them for performance. There are several models of VTs, from classical ones (Martins et al., 2004; Powell et al., 2004) to a recent one (Dulebohn and Hoch, 2017). Others analyze VTs at the management level (Hertel et al., 2005) and others analyze them as a systemic Input-Process-Output or IPO (Saldaña Ramos, 2010). This last model is based on others that studied face-to-face teams (Hoch and Kozlowski, 2014) and proposes adaptations to the model when studying VT.

**FIGURE 1:**
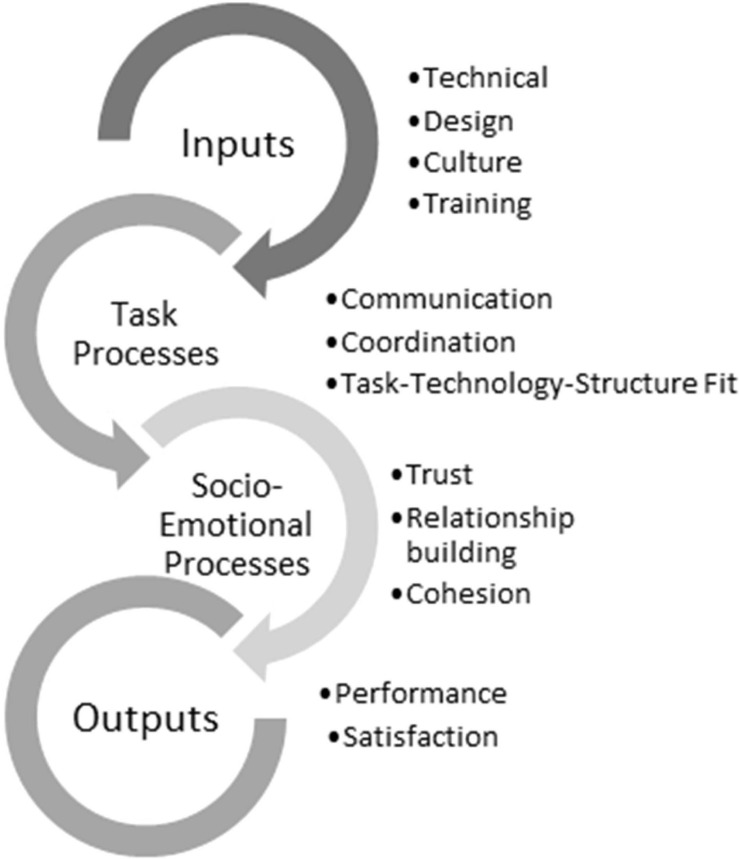
Reference IPO model for analyzing VTs. Source: Based on authors.

Research papers study the factors that influence VTs for virtual team management models and those that have a significant impact on performance are chosen and, in turn, are mentioned in the literature. As seen in Figure 1, this study has taken into account the different phases of the IPO model and its adaptation (Gilson et al., 2015) along with the factors that are organized into Inputs (related to communication and trust), Processes (task-oriented and socio-emotional) and Outputs (performance).

### Inputs

As observed in VT models, communication is studied in relation to the characteristics of the tasks that will be developed and co-created in a distributed way.

#### Task Features

The interaction between task type and communication and its impact on team performance has been investigated in the literature (Montoya-Weiss et al., 2001; Bell et al., 2002; Rico and Cohen, 2005). Because virtual teams rely heavily on communication technologies to coordinate their work, it is necessary to examine the relationship between the nature of the task and the effectiveness of communication that impacts team performance.

Software development projects are characterized by great uncertainty in terms of requirements and risk planning and followed by technological suitability until the project is completed. Task uncertainty has been conceptualized using various dimensions of task complexity in the literature. Some of the dimensions studied are task variety and task analyzability (Daft and Lengel, 1986); variability (de Ven et al., 1976); uniformity (Mohr, 1971); predictability (Galbraith, 1973); and complexity (Duncan, 1972). The proposed model of information processing by Daft and Macintosh (1981) is comprehensive and captures the nature of virtual teamwork effectively through the dimensions of task variety and task analyzability.

#### Trust

As seen in the VTs models, trust is considered as leadership, cohesion and team empowerment. These 3 characteristics are described in more detail below:

##### Leadership

One definition of leadership states that it is when a person gets other people to do something (Kort, 2008). Leadership is an influential relationship between leaders and followers who attempt to make changes that benefit their mutual purposes (Kort, 2008).

In VTs, transformational leadership seems to also arise from personality and communication factors (Balthazard et al., 2009) and can increase performance, satisfaction (Purvanova and Bono, 2009) and motivation (Andressen et al., 2012).

Clearly, leadership is important for VTs. In one study (Glückler and Schrott, 2007) it was found that communication influenced who emerged as a leader.

Glückler and Schrott (2007) found that communication behavior influenced who emerged as a leader. Similarly, leader–member exchange (Goh and Wasko, 2012), perceptions of supportive leadership (Schepers et al., 2011), leadership roles (Konradt and Hoch, 2007) and cross-cultural leadership (Sarker et al., 2009) have received attention, and other research has studied the impact of the type of recognition a leader uses to motivate workers (Whitford and Moss, 2009).

Research on VT leadership has grown rapidly, with two popular areas being leadership behavior and traits (Gilson et al., 2015). Here, the work has examined inspirational aspects (Joshi et al., 2009) as well as transformational and transactional leaders (Huang et al., 2010; David Strang, 2011). In VT, transformational leadership seems to be due to personality and communication factors (Balthazard et al., 2009) and can increase performance, satisfaction (Purvanova and Bono, 2009) and motivation (Andressen et al., 2012).

Several studies have examined the interaction between leadership and virtuality, finding that team members are more satisfied with their team and leader and perceive that their leader is better able to decode messages when the leader is geographically distant from the team (Henderson, 2008). Hoch and Kozlowski (2014) found that virtuality dampened the relationship between hierarchical leadership and performance while improving the relationship between structural supports and performance.

Clearly, leadership within VTs is important. As such, leaders can play a central role in how a VT works, particularly because they influence how a team deals with obstacles and how the team ultimately adapts to such challenges. This can be seen in articles on team adaptation research (Baard et al., 2014).

Other research suggests that classic leadership styles are appropriate for a virtual team:

Democratic (McBer and Company, 1980) and referee leadership styles (Rashid and Dar, 1994) have some characteristics that are very suitable for a virtual team. One negative factor could be that many meetings are needed to reach consensus. In a virtual team, it is difficult and time-consuming to hold meetings for each decision.

Operational leadership (McBer and Company, 1980) may be a good option because this leadership style gives team members clear roles and tasks. In addition, the leader makes the processes and structures very clear, so lack of communication will be reduced. A negative feature of this style of leadership for virtual teams might be that the contribution of the team members, and their responsibilities, might be a little less than the team members want.

Coaching leadership (McBer and Company, 1980) fits virtual teams very well because it gives a lot of freedom to the team members, which means that they are also responsible for their work and results. Team members can set their own goals and therefore also progress personally while working in the virtual team. This leadership style, however, also has some difficulties. The processes, structures and roles of the team may not always be very clear because the leader allows team members to establish and use their own. Therefore, the success of the virtual team might suffer a little.

##### Cohesion

According to Salisbury et al. (2006) research into classical teams (Lott and Lott, 1965; Hogg, 1987) suggest that the physical distance between members can be translated into a psychological distance between them. Following this line of reasoning (Salisbury et al., 2006) the physical dispersion of the virtual team could inhibit cohesion. In addition, virtual team members may have different ideas about what cohesion is. In other words, the idea of cohesion, which is the communication between group members, is affected by the medium used to communicate. This is especially true given the ease with which users can exchange non-task related information in some environments. Clearly, the differences in communication patterns between virtual and onsite teams suggest that measures (such as PCS) which are used in one context cannot be directly employed in another without reevaluating them (Boudreau et al., 2001).

Studies about group behavior (Hogg and Tindale, 2001) consistently report that, in working groups, the members’ ability to get along with each other is critical for well-being and task performance. The importance of developing such intra-group cohesion has been shown to be especially relevant in cases where members don not know each other, such as in newly formed groups or when members are assigned to new project teams (Griffin, 1997). The Symbolic Convergence Theory (SCT) proposed by Bormann (1983, 1996) and tested by Bormann et al. (1994, 1997) provides a rich theoretical framework for understanding group cohesion in traditional and technology-based teams.

One type of group cohesion is task cohesion and occurs when members stay together because they are strongly involved with the group’s tasks. Task cohesion will be greater if members identify with the group’s tasks and find them intrinsically rewarding and valuable.

Group cohesion for virtual teams with members working at different geographic locations, for different organizations, and even in different sectors of the economy, need effective communication and close coordination to achieve goals (Powell et al., 2004).

The positive relationship between cohesion and trust in working teams has been confirmed in many investigations (Evans and Dion, 1991; Simons and Peterson, 2000; Baltes et al., 2002; Powell et al., 2004; Spector, 2006; Lu, 2015).

##### Empowerment

Empowerment is favorable acknowledgment by the team leader and allows team members to participate in decision making. Empowerment makes the team member trust the leader, and when the leader asks for opinions and comments, he or she processes them and makes decisions based on the suggestions.

Some past studies (Kirkman et al., 2004) indicate that teams can be empowered in four different ways, (a) power, which is the collective belief that a team can be effective, (b) significance, which is the extent to which team members care about their tasks, (c) autonomy, in which team members have freedom to make decisions; and (d) impact, the degree to which team members feel that their tasks make important contributions.

The impact of team empowerment on the performance of EVTs in 10 telecommunications companies in Islamabad was studied by Gondal and Khan (2008). That study found that there is a positive relationship between team empowerment and team performance in telecommunications teams. Team performance includes the variables of cooperation, coordination, trust, cohesion, effort, mutual support, team conflict, job satisfaction and effectiveness in terms of quality.

Kirkman et al. (2004) also studied 35 sales and service teams at a high-tech firm and investigated the impact of team empowerment on team performance and the intermediary role of face-to-face interaction. They found that team empowerment is positively related to both constructs of virtual team performance, which are process improvement and customer satisfaction.

As indicated (Kirkman et al., 2004) empowerment in a virtual team can be a substitute for the leadership tasks of a single team leader (Kerr and Jermier, 1978). The behavior of the team members due to the leader’s empowerment is directly and positively related to trust. It is considered a confidence-building attribute. For empowerment, commitment is only reached when the team has a shared vision and honest and regular communication with the leader.

### Processes

Models usually study the processes of tasks by investigating communication and the social-emotional processes of trust. The degree of virtuality and the interrelationship of tasks are also considered important for performance.

#### Communication

In mixed teams, where some members are at the same physical location and others are not, communication problems can also occur. Team members at the same physical place often communicate in a deeper way than with the distant members and this ends up causing friction between them and, therefore, damages the performance of the team (Powell et al., 2004).

Communication, coordination and knowledge sharing are essential elements of action processes to predict the efficiency and effectiveness of the team (Kock and Lynn, 2012).

Another study (Peñarroja et al., 2013) found that as virtuality increased, team coordination declined, but this relationship was partially mediated by levels of trust.

Early research on VTs proposed that initial FtF meetings should help encourage performance (Geber, 1995). Han et al. (2011) extended this line of reasoning to creativity and compared modes of initial communication to assess their impact.

#### Trust

Understanding how, why, and under what conditions trust develops remains a popular research topic. In part, the importance of trust can be attributed to results that suggest it positively affects the success of VTs (Furumo, 2009).

For VTs, trust is influenced by communication behavior, timely responses, open communication, and feedback (Henttonen and Blomqvist, 2005).

More recent findings suggest that rapid trust is likely to be established with early communication and a positive tone (Coppola et al., 2004) and may influence performance by improving member confidence and subsequent trust (Crisp and Jarvenpaa, 2013).

Other research has studied the impact of global VTs on trust development (Lowry et al., 2010). Culturally heterogeneous teams (China and the United States) and homogeneous teams were compared and no significant differences were found in the trust between FtF teams and VTs (Lowry et al., 2010).

Furthermore, in a longitudinal study of global VTs, Goh and Wasko (2012) found that when everyone’s actions were visible, trust was not a key factor in resource allocation.

Finally, in globally distributed teams, trust mitigated the negative effects of member diversity on performance (Garrison et al., 2010).

### Output

Finally, aspects such as performance, quality of the product or service obtained and member satisfaction are relevant for the results. Of course, performance is the essential variable and is the usual interest of research into virtual teams.

#### Performance

Overall, research suggests that working in VTs can have a positive impact on effectiveness (Kock and Lynn, 2012; Maynard et al., 2012), while others provide evidence suggesting that virtual working affects effectiveness negatively (Cramton and Webber, 2005; Schweitzer and Duxbury, 2010).

A positive trend appears to be that work in this area is beginning to take advantage of ratings from outside the team (Andressen et al., 2012; Cummings and Haas, 2012), as well as objective measures of team performance (Rico and Cohen, 2005; Rapp et al., 2010).

In considering the elements of effectiveness, several researchers have examined the quality of the project (Altschuller and Benbunan-Fich, 2010). This makes sense, since VTs are often used for special projects. In addition, the quality of the decisions made and the time taken to reach a decision have been studied and the findings are often that VTs need more time to make decisions (Pridmore and Phillips-Wren, 2011).

Other studies find that VTs that set goals early in their life cycle showed greater cohesion and performance (Brahm and Kunze, 2012).

Other work in this area also suggests that team motivation and performance can be improved by using mixed incentive rewards (Bryant et al., 2009).

One study (Kirkman et al., 2013) considered the impact of national diversity on performance and found a curvilinear (U-shaped) relationship moderated by both media richness and psychological safety.

## Materials and Methods

The present study was carried out to understand the factors which influence the performance of VTs in a professional team that is used to using “agile” methodologies and virtual working.

A quantitative causal study using partial least squares (PLS) was performed using an online questionnaire, with a sample of 317 participants (Software Engineers).

### Questionnaire and Measurement Scales

A quantitative research divided into the following blocks was designed and then carried out and the results were used to test the hypotheses that constitute the theoretical model. The details are shown in Table 1.

**TABLE 1:** Variables of the proposed model.

**Variable**	**Definition**	**Authors**
Task characteristics	Represent elements of task uncertainty that have been the basis of many studies of organizational structure and process (Perrow, 1967)	Daft and Macintosh, 1981; Campion et al., 1993
VT communication	Defined as when group members must be able to clearly and explicitly exchange information to effectively support collaboration (Lowry et al., 2006).	Dennis and Kinney, 1998; Lowry et al., 2006; Makoul and Curry, 2007
Leadership	Defined as a dynamic process of social problem solving accomplished through generic responses to social problems (Burke et al., 2006)	Burke et al., 2006
Cohesion	Defined as the commitment of each team member to remain united in the pursuit of the team’s goals and to each member’s affective needs (Subramanyam, 2013).	Warkentin and Beranek, 1999; Wei et al., 2018
Empowerment	Defined as the collective belief in a group that it can be effective, and its role in determining group effectiveness (Guzzo et al., 1993).	Guzzo et al., 1993
Trust	Is a crucial factor in forming and maintaining social relationships and is key for cooperative relationships and effective teamwork (Alsharo et al., 2017)	Guzzo et al., 1993; De Jong and Elfring, 2010; Alsharo et al., 2017
Performance	Is the ability to work at the highest level of effectiveness for an extended period of time. This means delivering quality products on time, within budget, while satisfying stakeholders (Pitagorsky, 2007).	Fuller et al., 2006; Dayan and Di Benedetto, 2010; Alsharo et al., 2017

### Proposed Model

**FIGURE 2:**
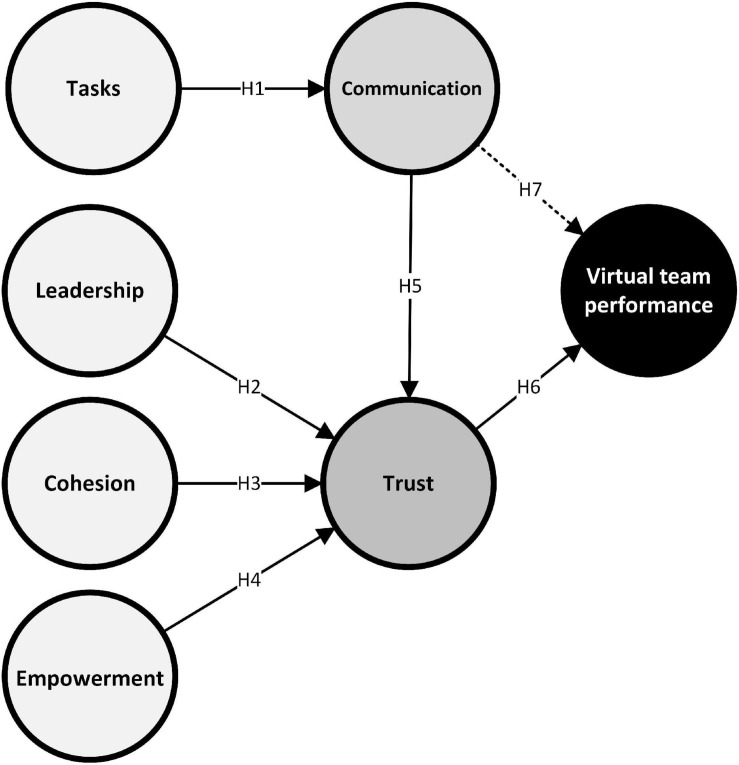
Proposed model.

The proposed model that incorporated the hypothetical relationships is illustrated in Figure 2.

### Research Hypotheses

The research hypotheses for the investigation of the factors that influence the performance of virtual teams are presented below.

#### Considerations of the Research Approach in the Hypotheses

Due to the quantitative approach chosen and by virtue of the delimiting nature of quantitative research, the hypotheses constitute the behavior that the variables or constructs are expected to show in the software development VT environment. Figure 2 shows the initial model. The hypotheses that are to be tested in this study are presented below:

H1: The characteristics of the tasks have a direct and positive influence on the communication of the virtual team members.

H2: The level of leadership of the members of the virtual team has a direct and positive influence on trust.

H3: The level of cohesion of the members of the virtual team has a direct and positive influence on trust.

H4: The level of empowerment of the members of the virtual team has a direct and positive influence on trust.

H5: Communication between virtual workers has a direct and positive influence on the confidence of the virtual team.

H6: Trust among virtual workers has a direct and positive influence on the performance of the virtual team.

H7: The level of communication between virtual workers has a direct and positive influence on the performance of the virtual team.

#### Hypothesis Research Scope Considerations

The correlational scope used to find the relationships between variables that give an answer to a problem means that without proving these relationships there could be a causal link between the variables. Figure 2 shows the constructs of the hypotheses in the study model.

Additionally, it is important to reiterate, that the VT performance construct is based on the relationships with the aggregate constructs Communication (h9) and Trust (h10) which in turn are expected to have a strong relationship between them and this will be tested in the research (h7 and h8). Then, the latent variable called communication has the constructs of cultural diversity (h1), the characteristics of the tasks (h2), as well as the distribution index (h3). Finally, the variables leadership (h4), cohesion (h5), and empowerment (h6) are used to find the latent variable trust.

The model used for the research hypotheses, its variables and its relationships are described in the literature review section.

### Sampling and Data Collection

1,200 software engineers with experience in programming with Agile methodology (which involves co-creation and collaboration in virtual teams) and who had graduated in the last 10 years, were directly invited to take part in the survey. 317 responses were collected.

## Results

### Strengths

The study was designed based on robust studies previously applied to telework and virtual teams in globally distributed teams for 20 years and after a robust literature review on the most relevant factors affecting the performance of these teams.

The study was applied at a privileged moment 3 months after the official declaration of the Covid pandemic19 by The World Health Organization.

The population taken into account for this study is considered stable because they were graduates of accredited engineering degrees from universities recognized in Costa Rica for their training in software development over the past 20 years and related colleagues.

Parallel to this study, a control study was conducted on another more heterogeneous population of professionals who in many cases had to start from scratch in the form of teleworking or virtual teams. This helped to understand and further refine the proposed model.

### Demographic Details

As can be seen in Table 2, the results found for the demographic features of the 317 members of virtual teams that use agile methodologies for the development of their projects are tabulated.

**TABLE 2:** Demographic details.

**Demographic details**	**Software engineering**
**Universe**	
n = 317	
%	100.00%
**Gender**	
Male	81.07%
Female	18.93%
**Age**	
18–29	64.98%
30–39	18.93%
40–49	10.41%
50–59	4.73%
60 or +	0.95%
**Time using VT**	
<1 year	58.99%
2–5 years	28.71%
6–10 years	7.57%
11–15 years	2.84%
16 or + years	1.89%
**Leader now**	
Leader	29.65%
Member	70.35%
**Leader before**	
Yes	58.04%
No	41.96%
**Same Organization**	
Yes	76.34%
No	23.66%
**Share Knowledge**	
Yes	65.93%
No	34.07%
**Future in VT**	
Yes	68.45%
No	2.84%
Maybe	28.71%

For gender, it is normal that in Software Engineering (SE) there is a higher proportion of men (81%) than women (19%). For age, it should be noted that 65% of those who responded to the questionnaire about virtual teams of SE were digital natives (born after the 1990s).

For the time spent working in VTs, almost 90% of the young members of SE VTs had joined in the last 5 years, which is consistent with handling agile methodologies and virtual teams in this profession.

The proportion of leaders is approximately 30% of the group and members 70%. In the SE VTs it was notable that 58% of the members have also been project leaders before, due to the dynamics of the Agile methodology and value co-creation. The diversity of membership in organizations shows that the members from SE VTs were 25% of the sample group and the members of VTs from other professions (OP) were 5% due to their recent incorporation into this way of working.

The members of SE VTs (68%) were very interested in continuing working in VTs in a new post-Covid19 normality.

### Important Findings

It is clear that the objective of the work is to analyze the determinants of performance in virtual teams in a time of pandemic, where conditions forced the vast majority of workers to develop their work within their homes remotely, forming virtual teams in which they already participated or had to organize in this way. With this objective, a survey has been conducted among software engineers and they have specified a structural equation model to analyze the relationship between different inputs and processes in the output. The results obtained show the relevance of communication and confidence in the performance of virtual teams. But before reviewing the complete model it is important to mention some important findings:

– The participants in this study were professionals in the area of computer science, dedicated to the development of software. Mainly digital natives with experience in VTs, people with ages between 18 and 29 years (64.98%) and digital migrants between 30 and 39 years (18.93%) with high mastery of information and communication technologies ICTs. In general, they consider that virtual teamwork is an excellent way to develop their work in the world of technology. It is part of their profession. In the worst case, some engineers maintain a neutral stance toward the issue of virtual teamwork. Under normal conditions they have worked in virtual mixed mode and face to face, so under 100% pandemic conditions, they really didn’t have much of an adjustment problem, because they were already doing it before. Even when asked about the future, a high number (68.45%) see themselves working in virtual teams and 28.71% in mixed mode.
– The professionals interviewed in many cases have indicated that communication in virtual teams is a factor that must be improved in frequency and quality because they feel that the initial instructions are not enough. Others take communication as a natural factor, regardless of whether the communication is virtual or face to face. Finally others indicate that communication in the virtual team is better with the good use of collaborative tools.
– Trust is a very important factor in the study, because it allows employees to perform their tasks at a distance in a better way, as long as their tasks are measured by objectives. Too many controls throughout the work process make the virtual collaborator feel watched and that he is being evaluated negatively.
– Regarding the geographical distribution, software engineers agree with professionals from other areas in that it saves them time and money and due to the intensive and natural use of ICT in their profession, the physical distance was not relevant to achieve the objectives.
– Regarding the cultural diversity in this study, being regional, the interviewees gave positive answers because the cultural differences did not influence their performance in the software development projects that have in common in a standardized way the computational language and the technological architectures.
– About the distribution of tasks, to be developed projects with agile methodologies, the specifications of functional and technical requirements are very clear from the beginning and also are clarified or refined in time with the coordination, co-creation and collaborative work, so engineers have clear what their tasks are throughout the process. As for the Interdependence of tasks there was no significant finding at the level of software development operations. It is possible that this is due to the fact that software projects are structured at the level of by-products and tasks in an orderly manner.
– By using agile methodologies to develop work with virtual teams and distributing tasks among members early on, empowering each member individually and in relation to others has been vital in software projects. Depending on the level of experience and individual skills, empowerment is increasingly important in virtuality.
– Leadership is a fundamental issue, which directly influences the confidence of virtual collaborators. In this study the members of the virtual teams gave it a moderate importance because of the work methodology and the mixed experience: virtual and face to face, the works are done in a collaborative and very horizontal way. Additionally, 58.04% indicated that they had already led some software development in this modality in the past.
– The virtual team software development has made the collaborators work longer interacting through the ICTs, fighting to achieve common objectives. This has made that the cohesion between them has increased at work level.

### Sample Frame

A random database of 1,000 software engineers graduated in the last 20 years from accredited software engineering or systems engineering careers at universities in Costa Rica, a country with a tradition and recognition of many years of software development for the region of Central and North America (mainly United States), was taken into account.

The survey was applied from May to July 2020, in the midst of the Covid19 pandemic, using an email invitation for respondents to fill out an electronic survey instrument using the Google Forms platform with 65 items.

### Limitations

There are many factors previously studied that influence in one way or another the performance of VTs, but at the level of the proposed model they cannot all be included because they have shown that their influence has not been very strong or because the type of population that was chosen for this specific study was not relevant. For example, a limitation of this study is that the dimension of rewards was not considered, since in recent similar studies they have not shown significant relationships (Tan et al., 2019).

A second limitation that could be considered, is related to the fact that, the respondents belong to different institutional environments, regularly projects of 5–10 members, in medium sized software development companies. In this sense, it is common that they use agile methodology as the project organization standard, which compensates for the differences in size of the parent organization, type of products developed, the member’s country of origin and the country of origin of the final client.

The cultural diversity that has been extensively studied in virtual teams, in this study was included in the survey but its results did not show a significant influence because the software development projects were usually regional and associated with the same continent and time zones with few differences.

## Analysis of Results

### Results for the Measurement Model

The measurement model was tested for internal reliability, convergent validity and discriminant validity. The internal reliability was evaluated using Cronbach’s alpha which needs a value of at least 0.70 for acceptable internal consistency (Hair et al., 2013). Causality was analyzed using indicator loadings. Composite reliability was also used to investigate causality (Werts et al., 1974). All the constructs had internal consistency as all the values for Cronbach’s alpha were higher than 0.7 (Fornell and Larcker, 1981; Bagozzi and Yi, 1988; Hair et al., 2011). Fornell and Larcker (1981) used the Average Variance Extracted (AVE) to assess convergent validity, and stated that an acceptable value for this factor is AVE ≥ 0.50.

Table 3 shows the element loads, Cronbach’s alpha and AVE which were found for the constructs. Values for Cronbach’s alpha ranged from 0.914 to 0.709, which is higher than the recommended level of 0.70 and therefore indicates strong internal reliability for the constructs. The composite reliability ranged between 0.946 and 0.837 and the AVE ranged between 0.632 and 0.853, which are higher than the recommended levels. The conditions for convergent validity were therefore met. The discriminant validity was calculated with the square root of the AVE and the cross-loading matrix. For satisfactory discriminant validity, the square root of the AVE of a construct should be greater than the correlation with other constructs (Fornell and Larcker, 1981).

**TABLE 3:** Reliability, validity of the constructs, Fornell–Larcker criterion and HTMT.

**Const**	**Alfa de Cronbach**	**CR**	**AVE**		**Fornell–Larcker criterion**						**HTMT**					
				**TASK**	**COH**	**COM**	**TRU**	**PER**	**EMP**	**LEAD**	**TASK**	**COH**	**COM**	**TRU**	**PER**	**EMP**
**TASK**	0.851	0.910	0.771	0.878												
**COH**	0.880	0.912	0.676	0.547	0.822						0.629					
**COM**	0.709	0.837	0.632	0.577	0.555	0.795					0.739	0.698				
**TRU**	0.864	0.902	0.648	0.599	0.786	0.615	0.805				0.698	0.898	0.781			
**PER**	0.914	0.946	0.853	0.487	0.523	0.439	0.696	0.924			0.550	0.579	0.540	0.776		
**EMP**	0.815	0.915	0.844	0.542	0.716	0.516	0.771	0.620	0.918		0.651	0.841	0.675	0.899	0.716	
**LEAD**	0.867	0.904	0.653	0.486	0.599	0.525	0.639	0.536	0.568	0.808	0.564	0.685	0.669	0.735	0.600	0.674

**CR, composite reliability; AVE, average variance extracted; COH, cohesion; COM, communication; TRU, trust; PER, performance; EMP, empowerment; and LEAD, leadership*.*

These researchers carried out simulation studies to demonstrate that a lack of discriminant validity is better detected by means of another technique called the heterotrait-monotrait ratio (HTMT), which they had discovered earlier. All the HTMT ratios for each pair of factors was <0.90.

### Results for the Structural Models

The structural model was built from the different relationships between the constructs. The hypotheses for the study were tested by analyzing the relationships between the different constructs in the model to see if they were supported (Chin and Newsted, 1999; Reinartz et al., 2009).

**FIGURE 3:**
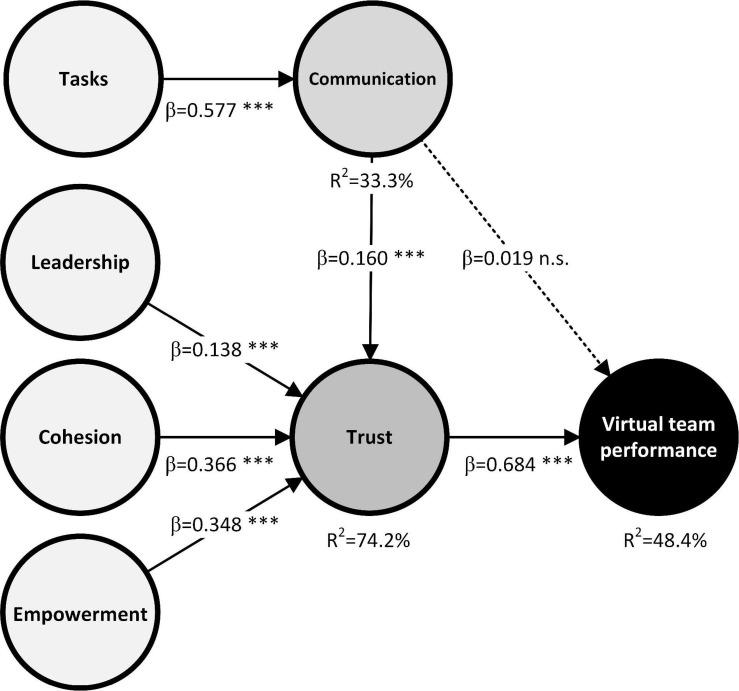
Final model. ***p < 0.001 [*t*(0.001; 499) = 3.106644601].

The variance is found from the values for the reflective indicators of the constructs (Barclay et al., 1995; Chin, 2010). This was found numerically by calculating the values of *R*^2^, which is a measure of the amount of variance for the construct in the model. The bootstrap method was used to test the hypotheses. The detailed results (path coefficient, β, and *t*-statistic) are summarized in Table 4 and Figure 3.

**TABLE 4:** Results of hypothesis: path coefficients and statistical significance.

**Hypothesis**	**β (Coeff. Path)**	**t statistic**	***p*-value**	**Supported**
H1 Characteristics of the tasks → communication of the members of the virtual team	0.577	13.842	0.000	Yes***
H2 Leadership in the members of the virtual teams → Trust	0.138	3.209	0.001	Yes***
H3 Cohesion in the members of the virtual teams → Trust	0.366	6.725	0.000	Yes***
H4 Empowerment for the members of the virtual teams → Trust	0.348	7.086	0.000	Yes***
H5 Communication between virtual workers → Trust	0.160	3.741	0.000	Yes***
H6 Trust among virtual workers → Performance of the virtual team	0.684	14.281	0.000	Yes***
H7 Communication between virtual workers → Performance of the virtual team	0.019	0.353	0.724	Not supported

*For n = 500 subsamples, using t distribution (499) of Students in a single queue.*

The measurements for approximate adjustments of the model (Henseler et al., 2016; Henseler, 2017) are given by the Standardized Root Mean Square Residual (SRMR) value (Hu and Bentler, 1998) which measures the difference between the observed correlation matrix and the implied correlation matrix of the model. SRMR shows the average magnitude of these differences.

A low value of SRMR means that the fit is better. In our case SRMR = 0.055, which was within the recommendations for a model with a good fit. A good fit is considered to be shown with a value of SRMR < 0.08 (Hu and Bentler, 1998).

The following conclusions were made from the values for *R*^2^ (see Table 5 and Figure 3) found in the research by Chin (1998) and show that 0.67 = “Substantial,” 0.33 = “Moderate,” and 0.19 = “Weak.” The result obtained for the main dependent variable of the model, Performance (PER) *R*^2^ = 48.4% was moderate and the rest of constructs, Trust *R*^2^ = 74.2% and Communication (COM) *R*^2^ = 33.3%.

**TABLE 5:** *R*^2^ results.

**Construct**	***R*^2^ (%)**
Communication (COM)	33.3
Trust (TRU)	74.2
Performance (PER)	48.4

This value shows that this model is “substantially” applicable to the performance of virtual teams. Please note that the variables that are not endogenous do not have a value for *R*^2^.

## Discussion

The results obtained for the proposed model have found that the performance of virtual teams is moderately justified by the determinants as *R*^2^ = 48.4%. However, the value obtained for Trust (*R*^2^ = 74.2%) should be noted as it means that the variance of this construct explains to a high percentage, aspects such as the confidence of the virtual team. This is essential to improve the co-creation of software development teams.

This study confirmed that the most significant variable for the performance of the EVT is Trust (H6), since this variable has the strongest influence on the dependent variable Performance. It also has a very high predictive capacity as the determination coefficient is high (β = 0.684; *t* = 14.281).

These results coincide with other recent findings that confirm that Trust can influence performance by improving member confidence and the subsequent trust (Crisp and Jarvenpaa, 2013). So when everyone’s actions are visible, trust was not a key factor in resource allocation (Goh and Wasko, 2012).

The next most important variable in the model is Task features (H1). Virtual teams rely heavily on communication technologies to coordinate their work, so the relationship between the nature of the task and the effectiveness of communication was studied in order to find its subsequent impact on team performance. Therefore, one of the determinants was the characteristics of the tasks and the positive influence on the communication of the members of the virtual team. The result was positive with a confidence level of 99.9%. Therefore, H1 was supported (β = 0.577; *t* = 13.842). These results amply confirm that great uncertainty about the requirements and the risk planning, followed by the technological suitability of the projects, are key to communication.

Our study also confirmed that the level of empowerment of the members of the virtual teams was also found to have a significant effect on Trust (H4). This result showed that Empowerment positively promotes and increases the confidence of a virtual team (β = 0.348; *t* = 7.086).

These results coincide with previous work (Gondal and Khan, 2008) that measured the impact of team empowerment on VT performance and demonstrated that there is a positive relationship between team empowerment and team performance in virtual teams. Our findings go further and state that this is achieved with Trust. As with other studies (Kirkman et al., 2004), empowerment in a virtual team can work as an alternative to leadership. Thus, the activities that are normally done by a team leader can be carried out by the members (Kerr and Jermier, 1978) by contributing with co-creation. This behavior of the team members because of the empowerment of the team members by the leader has a direct and positive relationship with trust. It is considered a confidence-building attribute. In empowerment, commitment is only reached when the team has a shared vision and honest and regular communication with the leader.

The relationship with the next highest confidence level for trust in the virtual teams was H3: the level of cohesion of the members of the virtual teams (β = 0.366; *t* = 6.725). This finding shows that the ability of the members of a virtual team to get along with each other is critical to the well-being of the group and task performance. These findings are consistent with previous work (Evans and Dion, 1991; Simons and Peterson, 2000; Baltes et al., 2002; Powell et al., 2004; Spector, 2006; Lu, 2015).

Therefore, it will be very important for software development companies to implement intragroup cohesion measures. These findings are consistent with other work (Griffin, 1997). Similarly, managers could implement economic incentives that support their software developers to be strongly involved with the group’s tasks. Task cohesion will be greater if members identify with the group’s tasks and find them intrinsically rewarding and valuable.

In the current context with the Covid-19 pandemic, this cohesion has been highly questioned. Let’s not forget that the isolation measures decreed by many governments have made it difficult to deal with aspects such as different geographical locations, belonging to different organizations, and different sectors of the economy. This has made effective communication and close coordination difficult. However, the results reaffirm the theories already shown (Powell et al., 2004).

One of the factors is the level of leadership of the members of the virtual teams (H2). The results showed that this had a direct and positive influence on Trust (β = 0.138; *t* = 3.209). Clearly, leadership in VTs is important. The results obtained coincide with the study by Baard et al. (2014) and show that the role of leaders is important for working in a VT, especially because leaders influence the way a team faces obstacles and the way the team ultimately adapts to such challenges, which is very important for the confidence generated for the future.

Therefore, the leader of a virtual team must use a style that generates Trust as a mediating factor in the indirect effect that this has on Performance.

The Communication between virtual workers has a direct and positive influence on the confidence of the virtual team and was supported (β = 0.160; *t* = 3.741) with a confidence level of 99.9%. Our study does support this hypothesis and agrees with Peñarroja et al. (2013), who found that as virtuality increased, team coordination declined, but this relationship was partially mediated by levels of Trust. In addition, as can be seen in the results, it is the least strongly supported hypothesis.

H7, the level of communication between virtual workers has a direct and positive influence on the performance of the virtual team, was not supported (β = 0.019; *t* = 0.353). This outcome appears to be conditioned by the very high levels of virtuality that have been reached during the containment measures decreed by governments at the start of the Covid-19 pandemic and, as stated above, clearly demonstrate that communication influences trust only through trust.

This result reaffirms the role of trust-building in achieving the highest performance of the virtual team and allows us to conclude that the confidence of all members in the virtual team is key to success in software development.

## Conclusion

The proposed model based on the IPO adaptation (Gilson et al., 2015) has been largely validated using a PLS-SEM analysis. Therefore, software companies can use it as a theoretical framework when preparing their human resources and Virtual Teams management policies.

The important role of Trust as a basis for most of the variables of the model shows that it should be considered as one of the most important and relevant variables, especially because of the increase in virtualization and teleworking during the Covid-19 pandemic. Companies must give greater importance to Trust and take into account that all measures which strengthen leadership, communication, cohesion or the configuration of task characteristics must be designed considering the trust generated. It is interesting to note that economic incentives can help with group cohesion and policies improve empowerment. One such incentive could be skills training for group members. These measures may become more important than leadership in the coming years, given the results found during the pandemic.

Finally, this study was completed with software developers who use agile methodologies and who have good IT skills. The results, therefore, show that the increased virtuality brought about by the pandemic can be an opportunity to innovate in communication to influence performance.

## Data Availability Statement

The raw data supporting the conclusions of this article will be made available by the authors, without undue reservation.

## Author Contributions

VG-A undertook the research, collected the data, and prepared the initial manuscript. PP-S completed, revised, and finalized the manuscript, and participated in the preparation of the manuscript. MA-C provided the intellectual input and analyzed the data. All authors contributed to the article and approved the submitted version.

## Conflict of Interest

The authors declare that the research was conducted in the absence of any commercial or financial relationships that could be construed as a potential conflict of interest.

## References

[B1] Abarca V. M. G. Palos-Sanchez P. R. Rus-Arias E. (2020). Working in virtual teams: a systematic literature review and a bibliometric analysis. *IEEE Access* 8 168923–168940. 10.1109/access.2020.3023546

[B2] Alsharo M. Gregg D. Ramirez R. (2017). Virtual team effectiveness: the role of knowledge sharing and trust. *Inf. Manage.* 54 479–490. 10.1016/j.im.2016.10.005

[B3] Altschuller S. Benbunan-Fich R. (2010). Trust, performance, and the communication process in ad hoc decision-making virtual teams. *J. Comput.Mediat. Commun.* 16 27–47. 10.1111/j.1083-6101.2010.01529.x

[B4] Andressen P. Konradt U. Neck C. P. (2012). The relation between self-leadership and transformational leadership: competing models and the moderating role of virtuality. *J. Leadersh. Organ. Stud.* 19 68–82. 10.1177/1548051811425047

[B5] Baard S. K. Rench T. A. Kozlowski S. W. J. (2014). Performance adaptation: a theoretical integration and review. *J. Manage.* 40 48–99. 10.1177/0149206313488210

[B6] Bagozzi R. P. Yi Y. (1988). On the evaluation of structural equation models. *J. Acad. Mark. Sci.* 16 74–94.

[B7] Baltes B. B. Dickson M. W. Sherman M. P. Bauer C. C. LaGanke J. S. (2002). Computer-mediated communication and group decision making: a meta-analysis. *Organ. Behav. Hum. Decis. Process.* 87 156–179. 10.1006/obhd.2001.2961

[B8] Balthazard P. A. Waldman D. A. Warren J. E. (2009). Predictors of the emergence of transformational leadership in virtual decision teams. *Leadersh. Q.* 20 651–663. 10.1016/j.leaqua.2009.06.008

[B9] Barclay D. Higgins C. Thompson R. (1995). The partial least squares (PLS) approach to casual modeling: personal computer adoption ans use as an Illustration. *Technol. Stud.* 2 285–309.

[B10] Bell M. Robertson D. Weeks M. Yu D. (2002). A virtual team group process. *Can. J. Nur. Leadersh.* 15 30–33. 10.12927/cjnl.2002.19157 12395975

[B11] Bormann E. G. (1983). “Symbolic convergence: organizational communication and culture,” in *Communication and Organizations: An Interpretive Approach*, eds Putnam L. Pacanowsky M. E., (Thousand Oaks, CA: SAGE Publications), 99–122.

[B12] Bormann E. G. (1996). Symbolic convergence theory and communication in group decision making. *Commun. Group Decis. Making* 2 81–113. 10.4135/9781452243764.n4

[B13] Bormann E. G. Craan J. F. Shields D. C. (1994). In defense of symbolic convergence theory: a look at the theory and its criticisms after two decades. *Commun. Theory* 4 259–294. 10.1111/j.1468-2885.1994.tb00093.x

[B14] Bormann E. G. Knutson R. L. Musolf K. (1997). Why do people share fantasies? An empirical investigation of a basic tenet of the symbolic convergence communication theory. *Commun. Stud.* 48 254–276. 10.1080/10510979709368504

[B15] Boudreau M.-C. Gefen D. Straub D. W. (2001). Validation in information systems research: a state-of-the-art assessment. *MIS Q.* 25 1–16. 10.2307/3250956

[B16] Brahm T. Kunze F. (2012). The role of trust climate in virtual teams. *J. Manage. Psychol.* 27 595–614. 10.1108/02683941211252446

[B17] Brett J. Behfar K. Kern M. C. (2006). *Managing Multicultural Teams.* Brighton, MA: Harvard Business Review. 17131565

[B18] Brooks S. K. Webster R. K. Smith L. E. Woodland L. Wessely S. Greenberg N. (2020). The psychological impact of quarantine and how to reduce it: rapid review of the evidence. *Lancet* 395 912–920. 10.1016/s0140-6736(20)30460-8 32112714 PMC7158942

[B19] Bryant S. M. Albring S. M. Murthy U. (2009). The effects of reward structure, media richness and gender on virtual teams. *Int. J. Account. Inf. Syst.* 10 190–213. 10.1016/j.accinf.2009.09.002

[B20] Burke C. S. Stagl K. C. Klein C. Goodwin G. F. Salas E. Halpin S. M. (2006). What type of leadership behaviors are functional in teams? A meta-analysis. *Leadersh. Q.* 17 288–307. 10.1016/j.leaqua.2006.02.007

[B21] Campion M. A. Medsker G. J. Higgs A. C. (1993). Relations between work group characteristics and effectiveness: implications for designing effective work groups. *Pers. Psychol.* 46 823–847. 10.1111/j.1744-6570.1993.tb01571.x

[B22] Chen C. de Rubens G. Z. Xu X. Li J. (2020). Coronavirus comes home? Energy use, home energy management, and the social-psychological factors of COVID-19. *Energy Res. Soc. Sci.* 68 101688. 10.1016/j.erss.2020.101688 32839705 PMC7341977

[B200] Chin W. W. (1998). The partial least squares aproach to structural equation modeling. *Mod. Methods Bus. Res.* 295, 295–336.

[B23] Chin W. W. (2010). “How to write up and report PLS analyses,” in *Handbook of Partial Least Squares*, eds Wang H. Henseler J. Vinzi V. E. Chin W. W., (Berlin: Springer), 655–690. 10.1007/978-3-540-32827-8_29

[B24] Chin W. W. Newsted P. R. (1999). Structural equation modeling analysis with small samples using partial least squares. *Stat. Strategies Small Sample Res.* 1 307–341.

[B25] Coppola N. W. Hiltz S. R. Rotter N. G. (2004). Building trust in virtual teams. *IEEE Trans. Prof. Commun.* 47 95–104. 10.1109/TPC.2004.828203

[B26] Cramton C. D. Webber S. S. (2005). Relationships among geographic dispersion, team processes, and effectiveness in software development work teams. *J. Bus. Res.* 58 758–765. 10.1016/j.jbusres.2003.10.006

[B27] Crisp C. B. Jarvenpaa S. L. (2013). Swift trust in global virtual teams. *J. Pers. Psychol.* 12 45–56. 10.1027/1866-5888/a000075

[B28] Cummings J. N. Haas M. R. (2012). So many teams, so little time: time allocation matters in geographically dispersed teams. *J. Organ. Behav.* 33 316–341. 10.1002/job.777

[B29] Daft R. L. Lengel R. H. (1986). Organizational information requirements, media richness and structural design. *Manage. Sci.* 32 554–571. 10.1287/mnsc.32.5.554 19642375

[B30] Daft R. L. Macintosh N. B. (1981). A tentative exploration into the amount and equivocality of information processing in organizational work units. *Adm. Sci. Q.* 26 207–224. 10.2307/2392469

[B31] David Strang K. (2011). Leadership substitutes and personality impact on time and quality in virtual new product development projects. *Proj. Manage. J.* 42 73–90. 10.1002/pmj.20208

[B32] Dayan M. Di Benedetto C. A. (2010). The impact of structural and contextual factors on trust formation in product development teams. *Ind. Mark. Manage.* 39 691–703. 10.1016/j.indmarman.2010.01.001

[B33] De Jong B. A. Elfring T. (2010). How does trust affect the performance of ongoing teams? The mediating role of reflexivity, monitoring, and effort. *Acad. Manage. J.* 53 535–549. 10.5465/amj.2010.51468649

[B34] de Ven A. H. Delbecq A. L. Koenig R. Jr. (1976). Determinants of coordination modes within organizations. *Am. Soc. Rev.* 41 322–338. 10.2307/2094477

[B35] Dennis A. R. Kinney S. T. (1998). Testing media richness theory in the new media: the effects of cues, feedback, and task equivocality. *Inf. Syst. Res.* 9 256–274. 10.1287/isre.9.3.256 19642375

[B36] Duarte D. L. Snyder N. T. (2006). *Mastering Virtual Teams: Strategies, Tools, and Techniques that Succeed.* Hoboken, NJ: John Wiley & Sons.

[B37] Dulebohn J. H. Hoch J. E. (2017). Virtual teams in organizations. *Hum. Resour. Manage. Rev.* 27 569–574. 10.1016/j.hrmr.2016.12.004

[B38] Duncan R. B. (1972). Characteristics of organizational environments and perceived environmental uncertainty. *Adm. Sci. Q.* 17 313–327. 10.2307/2392145

[B39] Ebrahim N. A. Ahmed S. Taha Z. (2009). Virtual teams: a literature review. *Aust. J. Basic Appl. Sci.* 3 2653–2669.

[B40] Evans C. R. Dion K. L. (1991). Group cohesion and performance: a meta-analysis. *Small Group Res.* 22 175–186. 10.1177/1046496491222002

[B41] Fornell C. Larcker D. F. (1981). Evaluating structural equation models with unobservable variables and measurement error. *J. Mark. Res.* 18 39–50. 10.2307/3151312

[B42] Fuller M. A. Hardin A. M. Davison R. M. (2006). Efficacy in technology-mediated distributed teams. *J. Manage. Inf. Syst.* 23 209–235. 10.2753/mis0742-1222230308

[B43] Furumo K. (2009). The impact of conflict and conflict management style on deadbeats and deserters in virtual teams. *J. Comput. Inf. Syst.* 49 66–73.

[B44] Galbraith J. R. (1973). *Designing Complex Organizations.* Boston, MA: Addison-Wesley Longman Publishing Co., Inc.

[B45] Garrison G. Wakefield R. L. Xu X. Kim S. H. (2010). Globally distributed teams: the effect of diversity on trust, cohesion and individual performance. *ACM SIGMIS Database Database Adv. Inf. Syst.* 41 27–48. 10.1145/1851175.1851178

[B46] Geber B. (1995). Virtual teams. *Training* 32 36–40.

[B47] Gilson L. L. Maynard M. T. Young N. C. J. Vartiainen M. Hakonen M. (2015). Virtual teams research: 10 Years, 10 themes, and 10 opportunities. *J. Manage.* 41 1313–1337. 10.1177/0149206314559946

[B48] Glückler J. Schrott G. (2007). Leadership and performance in virtual teams: exploring brokerage in electronic communication. *Int. J. E-Collaboration (IJeC)* 3 31–52. 10.4018/jec.2007070103

[B49] Goh S. Wasko M. (2012). The effects of leader-member exchange on member performance in virtual world teams. *J. Assoc. Inf. Syst.* 13 861–885. 10.17705/1jais.00308

[B50] Gondal A. M. Khan A. (2008). Impact of team empowerment on team performance: case of the telecommunications industry in Islamabad. *Int. Rev. Bus. Res. Papers* 4 138–146.

[B51] Griffin E. (1997). *Groupthink. A First Look at Communication Theory.* New York, NY: McGraw-Hill Education.

[B52] Guzzo R. A. Yost P. R. Campbell R. J. Shea G. P. (1993). Potency in groups: articulating a construct. *Br. J. Soc. Psychol.* 32 87–106. 10.1111/j.2044-8309.1993.tb00987.x 8467372

[B53] Hair J. F. Ringle C. M. Sarstedt M. (2011). PLS-SEM: indeed a silver bullet. *J. Mark. Theory Pract.* 19 139–152. 10.2753/mtp1069-6679190202

[B54] Hair J. F. Ringle C. M. Sarstedt M. (2013). Partial least squares structural equation modeling: rigorous applications, better results and higher acceptance. *Long Range Plan.* 46 1–12. 10.1016/j.lrp.2013.01.001

[B55] Han H.-J. Hiltz S. R. Fjermestad J. Wang Y. (2011). Does medium matter? A comparison of initial meeting modes for virtual teams. *IEEE Trans. Prof. Commun.* 54 376–391. 10.1109/tpc.2011.2175759

[B56] Henderson L. S. (2008). The impact of project managers’ communication competencies: validation and extension of a research model for virtuality, satisfaction, and productivity on project teams. *Proj. Manage. J.* 39 48–59. 10.1002/pmj.20044

[B57] Henseler J. (2017). Bridging design and behavioral research with variance-based structural equation modeling. *J. Adv.* 46 178–192. 10.1080/00913367.2017.1281780

[B58] Henseler J. Hubona G. Ray P. A. (2016). Using PLS path modeling in new technology research: updated guidelines. *Ind. Manage. Data Syst.* 116 2–20. 10.1108/imds-09-2015-0382

[B59] Henttonen K. Blomqvist K. (2005). Managing distance in a global virtual team: the evolution of trust through technology-mediated relational communication. *Strategic Change* 14 107–119. 10.1002/jsc.714

[B60] Hertel G. Geister S. Konradt U. (2005). Managing virtual teams: a review of current empirical research. *Hum. Resour. Manage. Rev.* 15 69–95. 10.1016/j.hrmr.2005.01.002

[B61] Hoch J. E. Kozlowski S. W. J. (2014). Leading virtual teams: hierarchical leadership, structural supports, and shared team leadership. *J. Appl. Psychol.* 99 390–403. 10.1037/a0030264 23205494

[B62] Hogg M. A. (1987). “Social identity and group cohesiveness,” in *Rediscovering the Social Group: A Self-Categorization Theory*, ed. Turner J., (New York, NY: Basil Blackwell), 89–116.

[B63] Hogg M. A. Tindale R. S. (2001). *Group Processes.* Malden, MA: Blackwell.

[B64] Hu L. Bentler P. M. (1998). Fit indices in covariance structure modeling: sensitivity to underparameterized model misspecification. *Psychol. Methods* 3:424. 10.1037/1082-989x.3.4.424

[B65] Huang R. Kahai S. Jestice R. (2010). The contingent effects of leadership on team collaboration in virtual teams. *Comput. Hum. Behav.* 26 1098–1110. 10.1016/j.chb.2010.03.014

[B66] Jarrahi M. H. Sawyer S. (2013). Social technologies, informal knowledge practices, and the enterprise. *J. Organ. Comput. Electron. Commer.* 23 110–137. 10.1080/10919392.2013.748613

[B67] Joshi A. Lazarova M. B. Liao H. (2009). Getting everyone on board: the role of inspirational leadership in geographically dispersed teams. *Organ. Sci.* 20 240–252. 10.1287/orsc.1080.0383 19642375

[B68] Kerr S. Jermier J. M. (1978). Substitutes for leadership: their meaning and measurement. *Organ. Behav. Hum. Perf.* 22 375–403. 10.1016/0030-5073(78)90023-5

[B69] Kirkman B. L. Cordery J. L. Mathieu J. Rosen B. Kukenberger M. (2013). Global organizational communities of practice: the effects of nationality diversity, psychological safety, and media richness on community performance. *Hum. Relations* 66 333–362. 10.1177/0018726712464076

[B70] Kirkman B. L. Rosen B. Tesluk P. E. Gibson C. B. (2004). The impact of team empowerment on virtual team performance: the moderating role of face-to-face interaction. *Acad. Manage. J.* 47 175–192. 10.5465/20159571 20159571

[B71] Kock N. Lynn G. S. (2012). Electronic media variety and virtual team performance: the mediating role of task complexity coping mechanisms. *IEEE Trans. Prof. Commun.* 55 325–344. 10.1109/TPC.2012.2208393

[B72] Konradt U. Hoch J. E. (2007). A work roles and leadership functions of managers in virtual teams. *Int. J. E-Collaboration (IJeC)* 3 16–35. 10.4018/jec.2007040102

[B73] Kort E. D. (2008). What, after all, is leadership?‘Leadership’and plural action. *Leadersh. Q.* 19 409–425. 10.1016/j.leaqua.2008.05.003

[B74] Lin C. Standing C. Liu Y.-C. (2008). A model to develop effective virtual teams. *Decis. Support Syst.* 45 1031–1045. 10.1016/j.dss.2008.04.002

[B75] Lott A. J. Lott B. E. (1965). Group cohesiveness as interpersonal attraction: a review of relationships with antecedent and consequent variables. *Psychol. Bull.* 64:259. 10.1037/h0022386 5318041

[B76] Lowry P. B. Roberts T. L. Romano N. C. Jr. Cheney P. D. Hightower R. T. (2006). The impact of group size and social presence on small-group communication: does computer-mediated communication make a difference? *Small Group Res.* 37 631–661. 10.1177/1046496406294322

[B77] Lowry P. B. Zhang D. Zhou L. Fu X. (2010). Effects of culture, social presence, and group composition on trust in technology-supported decision-making groups. *Inf. Syst. J.* 20 297–315. 10.1111/j.1365-2575.2009.00334.x

[B78] Lu L. (2015). Building trust and cohesion in virtual teams: the developmental approach. *J. Organ. Eff. People Perf.* 2 55–72. 10.1108/JOEPP-11-2014-0068

[B79] Makoul G. Curry R. H. (2007). The value of assessing and addressing communication skills. *Jama* 298 1057–1059. 10.1001/jama.298.9.1057 17785653

[B80] Martinez-Cañas R. Ruiz-Palomino P. Linuesa-Langreo J. Blázquez-Resino J. J. (2016). Consumer participation in co-creation: an enlightening model of causes and effects based on ethical values and transcendent motives. *Front. Psychol.* 7:793. 10.3389/fpsyg.2016.00793 27303349 PMC4880595

[B81] Martins L. L. Gilson L. L. Maynard M. T. (2004). Virtual teams: what do we know and where do we go from here? *J. Manage.* 30 805–835. 10.1016/j.jm.2004.05.002

[B82] Maynard M. T. Mathieu J. E. Rapp T. L. Gilson L. L. (2012). Something(s) old and something(s) new: modeling drivers of global virtual team effectiveness. *J. Organ. Behav.* 33 342–365. 10.1002/job.1772

[B83] McBer and Company. (1980). *Trainer’s Guide.* Boston, MA: McBer and Company.

[B84] Mohr L. B. (1971). Organizational technology and organizational structure. *Adm. Sci. Q.* 16 444–459. 10.2307/2391764

[B85] Montoya-Weiss M. M. Massey A. P. Song M. (2001). Getting it together: temporal coordination and conflict management in global virtual teams. *Acad. Manage. J.* 44 1251–1262. 10.2307/3069399

[B86] Palos P. R. Correia M. B. (2017). La actitud de los recursos humanos de las organizaciones ante la complejidad de las aplicaciones SaaS. *Dos Algarves Multidiscip. J.* 28 87–103. 10.18089/damej.2016.28.1.6

[B87] Palos-Sanchez P. R. (2017). El cambio de las relaciones con el cliente a través de la adopción de APPS: estudio de las variables de influencia en M-Commerce. *Rev. Espacios* 38:38.

[B88] Peñarroja V. Orengo V. Zornoza A. Hernández A. (2013). The effects of virtuality level on task-related collaborative behaviors: the mediating role of team trust. *Comput. Hum. Behav.* 29 967–974. 10.1016/j.chb.2012.12.020

[B89] Perrow C. (1967). A framework for the comparative analysis of organizations. *Am. Soc. Rev.* 32 194–208. 10.2307/2091811

[B90] Piccoli G. Powell A. Ives B. (2004). Virtual teams: team control structure, work processes, and team effectiveness. *Inf. Technol. People* 17 359–379. 10.1108/09593840410570258

[B91] Pitagorsky G. (2007). “Managing virtual teams for high performance,” in *Paper Presented at PMI§Global Congress*, (North America, Atlanta, GA: Project Management Institute).

[B92] Powell A. Piccoli G. Ives B. (2004). Virtual teams: a review of current literature and directions for future research. *SIGMIS Database* 35 6–36. 10.1145/968464.968467

[B93] Pridmore J. Phillips-Wren G. (2011). Assessing decision making quality in face-to-face teams versus virtual teams in a virtual world. *J. Decis. Syst.* 20 283–308. 10.3166/jds.20.283-308

[B94] Purvanova R. K. Bono J. E. (2009). Transformational leadership in context: Face-to-face and virtual teams. *Leadersh. Q.* 20 343–357. 10.1016/j.leaqua.2009.03.004

[B95] Rapp A. Ahearne M. Mathieu J. Rapp T. (2010). Managing sales teams in a virtual environment. *Int. J. Res. Mark.* 27 213–224.

[B96] Rashid M. Dar J. (1994). Current managerial styles & effective managers. *Manage. Serv.* 38 16–17.

[B97] Reinartz W. Haenlein M. Henseler J. (2009). An empirical comparison of the efficacy of covariance-based and variance-based SEM. *Int. J. Res. Mark.* 26 332–344. 10.1016/j.ijresmar.2009.08.001

[B98] Ribes-Giner G. Perelló-Marin M. R. Pantoja-Diaz O. (2017). Revisión sistemática de literatura de las variables clave del proceso de co-creación en las instituciones de educación superior. *Tec. Empre.* 11 41–53. 10.18845/te.v11i3.3365

[B99] Rico R. Cohen S. G. (2005). Effects of task interdependence and type of communication on performance. *J. Manage. Psychol.* 20 261–274. 10.1108/02683940510589046

[B100] Saldaña Ramos J. (2010). *VTManager: Un Marco Metodológico Para la Mejora de la Gestión de Los Equipos de Desarrollo Software Global.* Madrid: Universidad Carlos III de Madrid.

[B101] Salisbury W. D. Carte T. A. Chidambaram L. (2006). Cohesion in virtual teams: validating the perceived cohesion scale in a distributed setting. *SIGMIS Database* 37 147–155. 10.1145/1161345.1161362

[B102] Sánchez P. R. P. (2017). Drivers and barriers of the cloud computing in SMEs: the position of the European union. *Harv. Deusto Bus. Res.* 6 116–132.

[B103] Sarker S. Sarker S. Schneider C. (2009). Seeing remote team members as leaders: a study of US-Scandinavian teams. *IEEE Trans. Prof. Commun.* 52 75–94. 10.1109/TPC.2008.2007871

[B104] Schepers J. de Jong A. de Ruyter K. Wetzels M. (2011). Fields of gold: perceived efficacy in virtual teams of field service employees. *J. Service Res.* 14 372–389. 10.1177/1094670511412354

[B105] Schweitzer L. Duxbury L. (2010). Conceptualizing and measuring the virtuality of teams. *Inf. Syst. J.* 20 267–295. 10.1111/j.1365-2575.2009.00326.x

[B106] Shuffler M. L. Wiese C. W. Salas E. Burke C. S. (2010). Leading one another across time and space: exploring shared leadership functions in virtual teams. *Rev.Psicolog Trabajo Las Organ.* 26 3–17. 10.5093/tr2010v26n1a1

[B107] Simons T. L. Peterson R. S. (2000). Task conflict and relationship conflict in top management teams: the pivotal role of intragroup trust. *J. Appl. Psychol.* 85:102. 10.1037/0021-9010.85.1.102 10740960

[B108] Spector T. (2006). Does the sustainability movement sustain a sustainable design ethic for architecture? *Environ. Ethics* 28 265–283. 10.5840/enviroethics200628317

[B109] Subramanyam V. (2013). Team cohesion between national youth and junior volley ball players: a comparative analysis. *Int. J. Sports Sci. Fitness* 3, 250–258.

[B110] Tan C. K.\ Ramayah T. Teoh A. P. Cheah J.-H. (2019). Factors influencing virtual team performance in Malaysia. *Kybernetes* 48, 2065–2092. 10.1108/K-01-2018-0031

[B111] Velicia-Martin F. Cabrera-Sanchez J.-P. Gil-Cordero E. Palos-Sanchez P. R. (2021). Researching COVID-19 tracing app acceptance: incorporating theory from the technological acceptance model. *PeerJ Comput. Sci.* 7:e316. 10.7717/peerj-cs.316 PMC7924669 33816983

[B112] Warkentin M. Beranek P. M. (1999). Training to improve virtual team communication. *Inf. Syst. J.* 9 271–289. 10.1046/j.1365-2575.1999.00065.x

[B113] Wei L. H. Thurasamy R. Popa S. (2018). Managing virtual teams for open innovation in Global Business Services industry. *Manage. Decis.* 56 1285–1305. 10.1108/MD-08-2017-0766

[B114] Werts C. E. Linn R. L. Jöreskog K. G. (1974). “Quantifying unmeasured variables,” in *Measurement in the Social Sciences*, ed. Blalock H. M., (Chicago: Aldine Publishing Co), 270–292. 10.4324/9781351329088-11

[B115] Whitford T. Moss S. A. (2009). Transformational leadership in distributed work groups: the moderating role of follower regulatory focus and goal orientation. *Commun. Res.* 36 810–837. 10.1177/0093650209346800

[B116] Zúñiga Ramirez C. Solano Cordero J. Bolaños Garita R. (2016). Quantic trends in knowledge-based companies: a case analysis of a Costa Rican experience. *Tec. Empresarial* 10 29–40. 10.18845/te.v10i3.2938

